# Assessment of care provision integration in a community-based mental health system: balanced care model implementation in Andalusia (Spain)

**DOI:** 10.1186/s12889-024-20169-6

**Published:** 2024-09-30

**Authors:** Diego Diaz-Milanes, Nerea Almeda, Maria Luisa Rodero-Cosano, Jose A. Salinas-Perez, Carlos R. Garcia-Alonso

**Affiliations:** 1 Department of Quantitative Methods, Universidad Loyola Andalucia, Seville, Spain; 2 Health Research Institute, University of Canberra, Canberra, ACT Australia; 3 Department of Psychology, Universidad Loyola Andalucia, Seville, Spain

**Keywords:** Mental health services, Health care system assessment, Balanced care model, Decision support system, Relative technical efficiency, Cartographic representation

## Abstract

**Background:**

Andalusia is the second largest region in Spain, and it has developed a comprehensive mental health (MH) plan that encourages the consolidation of the balanced care model. However, its geographical and socioeconomic disparity is a great challenge for a community-based MH system. Both the assessment of the implementation of the MH plan and the development of new tools to support decision-making can be considered critical.

**Objectives:**

The present study aims (i) to assess how the integration of different types of MH care may influence system performance and (ii) to check the performance evolution of the integration process geographically regarding the small MH areas of Andalusia.

**Methods:**

The performance of the Andalusian MH system was assessed by combining Monte Carlo simulation, fuzzy inference and data envelopment analysis. The relative technical efficiency was the main performance indicator.

**Results:**

A correct integration of appropriate types of MH care, according to population needs, increases the performance of the Andalusian MH system both from global and regional perspectives. The spatial representation (based on small MH areas) of the results highlights how the performance depends on specific geographical characteristics. By analyzing the identified spatial clusters, defined by different management patterns depending on user and socioeconomic characteristics, benchmark areas and areas for improvement can be studied to design evidence-informed policies and interventions.

**Conclusions:**

A global analysis of MH system performance was carried out, including both the successive integration of different types of care and its spatial evolution. Although an appropriate integration of different types of MH care has a positive effect on the Andalusian MH system, this process has different profiles depending on specific geographically based user and socioeconomic characteristics. The balanced care model can be considered the paradigm for assessing the performance of a large and populated territory such as Andalusia, which has a community-based MH system. This methodological approach (performance assessment and spatial analysis) may be used as a guide for developing future evidence-informed policies and managerial interventions.

**Supplementary Information:**

The online version contains supplementary material available at 10.1186/s12889-024-20169-6.

## Background

Currently, mental disorders are one of the biggest public health problems, especially after the COVID-19 pandemic [1]. In 2019, it was estimated that one in eight people globally suffered from a mental health (MH) disorder, amounting to 970 million individuals, with anxiety and depression-related disorders being the most common [2]. The WHO European Framework for Action on Mental Health 2021–2025 [3] estimates that the number of people with MH problems stands at more than 125 million, equivalent to 13% of the population of the European Region in 2019, and represents an adjusted disability level of 15% life years. Furthermore, depressive disorders are expected to become an even more significant cause of global disability-adjusted life years by 2050 [4].

At the same time, the services, skills, and funding available for MH remain scarce and fall far short of what is needed worldwide [5]. For this reason, the main objectives of the Pan-European MH Coalition reflect the three key priorities: transforming MH services, integrating MH into emergency response and recovery plans, and promoting and protecting MH throughout the life course [3].

In line with these objectives, the MH strategy of Spain 2022–2026 states that MH care must turn toward a person-centered care approach, which implies providing a role in the recovery process to the person who has experienced MH problems [6]. However, significant disparities in services provision can be observed across the country [7]. For example, in 2010, it was still possible to find a public system without the closure of psychiatric hospitals, such as the Basque Country, while Andalusia had a full closure of them [7].

Andalusia possesses a comprehensive MH plan that encourages the consolidation of the community care model in its different areas of action. This model promotes the process of transforming MH care toward greater effectiveness and efficiency through expanding complementary and inclusive networking of the different types of professionals and sectors involved [8]. This is in line with the balanced care model promoted by the WHO, which is community-based, integrated by multidisciplinary MH teams and supported by experts in primary health care acting as a gate to the MH services [3], and actively seeks a pragmatic balance between all of the service components that are present in the system, according to the corresponding socioeconomic structure [9, 10].

Because of Andalusia’s characteristics, the adequate implementation of the abovementioned model is a priority. This is the second largest region in Spain, occupying 17.3% of its territory, and the most populated, containing 18% of its population. According to the PISMA-ep [9] study, 15% of the population meets the criteria for a mental disorder [8], and the dispensation of antidepressants increased for the 2000–2010 decade [10]. From a geographical point of view, it is a very heterogeneous territory with significant differences between urban and rural areas [11]. Only 1.5% of Andalusian municipalities have more than 100,000 inhabitants, and more than 25% have less than 1,000 inhabitants. This geographical disparity and difficulty of access is one of the most critical characteristics concerning the provision of services, especially in the most deprived areas, which are located in rural zones with poor accessibility [12].

Previous studies focused on the Andalusian MH system have been carried out to identify and describe the patterns of care provided by the social care sector [13] or to assess the distribution of MH care and case load in the region [11]. However, none of them has considered the relationship between care provision and the utilization of services according to the balanced care model. For a deep understanding of the relationships among the different components of a MH system, progress has been made in developing tools and instruments for technical support. To do so, collaborating with local experts and decision-makers is crucial to ensure the relevance and meaning of the obtained results and to develop appropriate evidence-informed policies [14].

This perspective, named the ecosystem approach, shifts away from a reductionist approach that focuses on creating simple answers to complex issues. This standpoint examines the environment and context and develops decision-support tools to help policy-makers [14]. This comprehensive approach to scientific knowledge includes local, expert, and professional knowledge and experimental, observational, and local evidence for health systems research [14, 15].

This approach has been shown to be suitable for assessing the performance of MH systems in northern Spain [16], including the complex relationships among types of care and facilities, as well as advanced methodologies for health care system assessment [17].

Due to the high level of interactions and components considered, data visualization tools are necessary for a correct interpretation of the results [18]. Although some of those studies have already proposed graphical representations to explain the behavior and performance of different MH areas [9, 12, 19], none has linked it to their geographical projection. The integration of geographical elements in interpreting health care administrative data is essential for determining the provision and location of health care services in relation to needs [20]. For that reason, more studies should focus on effective ways of using visual analytics to support decision-makers and administrators to better understand the performance of their MH systems or service networks, gaps in care or outcome measurements [18, 21].

In response to the gaps mentioned above, an assessment of the performance of the Andalusian MH areas regarding the integration of different types of care is proposed with a two-fold approach: the Decision Support System (DSS) Efficient Decision Support-MH (EDeS-MH; 23) was utilized to evaluate the performance of each area. This DSS includes Monte Carlo simulation to integrate the uncertainty of the analysed healthcare system (raw data was transformed into statistical distributions) and for increasing the number of units (DMU) artificially, a fuzzy inference engine that allows the interpretation and transformation of the simulated values according to the knowledge-base structured by IF…THEN… rules, and finally, the processed data is analysed by a Data Envelopment Analysis (DEA) model in order to assess the performance of the MH system according to the integration of different types of care. The EDeS-MH tool has previously been used to assess the effectiveness of MH systems at various levels of management, including macro and meso levels in Spain [22, 23], and micro levels in countries such as England [24] and Finland [25]. Additionally, it has also used for evaluating interventions associated to MH system management [16, 26].

These methods enable the assessment of the implementation of the balanced care model in a real community-oriented MH system. Therefore, the aims of this study are: (i) to assess how the integration of different types of MH care may influence system performance, and (ii) to check the performance evolution of the integration process geographically by dividing the Andalusian territory into small MH areas. The results of this study may be used to identify potential geographical areas for benchmarking, as well as those to be improved.

## Materials and methods

### Area of study

The autonomous region of Andalusia has complete control over its public health care provision through the Andalusian Health Service. The Andalusian Health Service provides care to a population of close to 8 and a half million people. Primary care services detect and refer severe cases of MH disorders to the specialized MH service network. MH services offer specialized and comprehensive care in the community throughout the region [27]. This network includes services such as outpatient care, health day care, rehabilitation services, hospital services, and therapeutic communities, all of which are closely related to the social care network for severe cases, named the Andalusian Public Foundation for the Social Integration of People with Mental Illness (FAISEM by its acronym in Spanish) [28].

### Procedure for data acquisition and pre-processing

#### Services description and classification

**Fig. 1:**
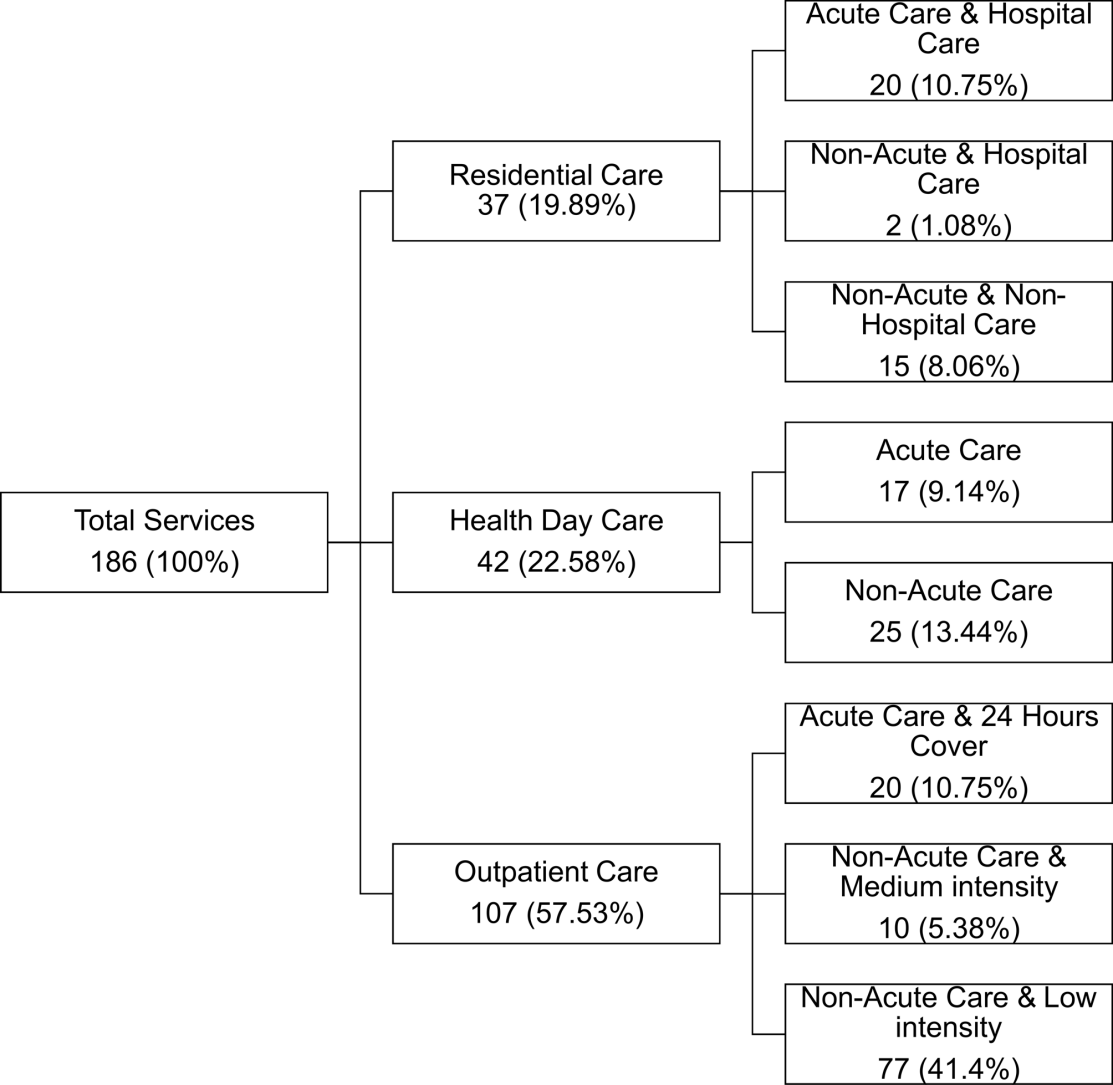
Public MH services distribution for adults population in Andalusia

Data about services, workforces and resources were drawn from the service mapping carried out in the REFINEMENT España project (2016–2018), which was the Spanish national continuation of the European Union-funded REFINEMENT project (REsearch on FINancing systems’ Effect on the quality of MENTal health care). The description of the services provided can be found in Fig. 1. The details and location of each MH care unit were extracted from the public health services repository [29] and cross-validated with the data obtained from the REFINEMENT project and the Andalusian Regional Government. Therefore, this research was focused on the public MH care provided by the specialized MH service network to the adult population of the region.

Regarding the description and care provided each of the MH services was classified by using the Description and Evaluation of Care Services and Directories for Long Term Care (DESDE-LTC) [30–32]. It was used to describe the public specialized MH service network of Andalusia through a standardized coding system. The DESDE-LTC is an international classification system for categorizing care units and service availability, allowing for international and cross-jurisdictional comparisons. The instrument is divided into main branches according to the care or service provided by Care teams or Basic Stable Inputs of Care presented in the tool, such as Residential (“R”), Day (“D”), or Outpatient (“O”) care, among others. In addition, an international glossary of terms was employed to solve terminology and comparability issues in MH ecosystem research [33].

In order to assess the integration of the main MH care services that should provide and/or support directly the care in the community, outpatient, acute hospital residential, and healthcare services were considered. However, a specific service can provide more than one type of care. In such cases, two different codes were applied, as with the acute hospital services with 24-hour physician cover, which provided both outpatient and residential care. A explanation of service typology, characteristics, codes and examples can be found in Table 1.

**Table 1:** Service types and code according to DESDE‑LTC

Main type of care	Service characteristics	DESDELTC code	Examples of facilities
Outpatient care	Nonacute, nonmobile, low intensity	O10	Community MH teams, outpatient psychiatric clinics and singlehanded psychiatrists and psychologists
	Nonacute, nonmobile, medium intensity	O9	
	Acute, nonmobile, 24 h physician cover	O3	General hospitals, psychiatric hospitals and other specialized hospitals
Residential care	Acute, hospital, 24 h physician cover, medium intensity care	R2	
Health day care	Acute, continuous care	D1	Day hospital
	Non Acute, Non-work structured, High intensity, Health related	D4.1	Day care center

#### Catchment area identification and calculation rates

In order to follow an ecosystemic approach, the MH service network provided by the Andalusian Health Service was analysed at the level of small health catchment areas (SHAs). The Andalusian MH network is territorially organized in 77 SHAs. All of them have a MH center that provides outpatient care and is considered the “reference”; other services and types of care can be integrated in the same SHA, such as health day care facilities or acute hospital care, among others. Both SHA borders and their corresponding MH services were georeferenced and linked to their variables and performance indicators [34]. Some of the Andalusian capitals (Seville, Granada, Malaga, Cordova and Jaen) have more than one “reference” center. However, the exact distribution of the users and the borders inside the urban area are not completely clear. For that reason, urban SHAs were merged into only one. The total number of analyzed SHAs was 65, which comprised the study units.

For this research within each SHA, two types of variables were selected, namely, structural variables related to the service capacity and service utilization variables. The first type consisted of service availability, placement capacity and workforce capacity. These variables were transformed into rates per 100,000 inhabitants (adults equal to or over 18 years old). The second type consisted of treated prevalence and frequentation; again, these variables were transformed into rates per 1,000 inhabitants (adults equal to or over 18 years old). According to the methodology, the structural variables were considered the input set (resource availability), and the prevalence and frequentation variables were considered the output set (outcome proxies). The dataset and the detailed description of each variable used in this study are available at datasheet [see Additional file 1].

### Analysis tool: data interpretation and analysis

An adaptation of the hybrid Decision Support System EDeS-MH (Efficient Decision Support-MH) was utilized to evaluate the performance of each SHA by analyzing input variables (resource availability) and output variables (resource outcomes) [26]. This computer-based tool incorporates a Monte Carlo simulation engine, a fuzzy inference engine, the design of analysis scenarios, and Data Envelopment Analysis (DEA). It was employed to process the data and conduct the analyses. The methodology and tool implemented in this study have been previously utilized for analyzing the performance of other regional and national MH systems, as extensively described in prior research [16, 22, 26, 35].

#### Monte Carlo simulation engine

The simulation engine was developed to include the inner uncertainty and randomness of the real system by artificially multiplying the number of observations [36]. Each piece of raw data in the original database was transformed into a statistical distribution, going from a standard dataset to a statistical distribution dataset. The Monte Carlo simulation engine reproduced these statistical distributions randomly for each SHA. This process stopped when the statistical error was lower than 2.5% on average [37].

#### Fuzzy inference engine

The fuzzy inference engine was used to interpret each variable value (from the Monte Carlo simulation engine) by using IF… THEN… rules based on the balanced care model [38–40]. The set of rules has been implemented in previous MH care performance studies developed in Spain [16, 22, 24, 26]. These rules activate the appropriate mathematical functions for the transformation (linear monotone increasing/decreasing) of the original variable values. These mathematical transformations allow the DSS to understand whether a specific variable value can be considered “appropriate” or not (at a certain level) according to the balanced care model used as the management paradigm. For example, a rate of 3.15 places/100,000 adults can be considered appropriate or not according to the balanced care model and expert knowledge. If it is considered appropriate (or not), the original value is transformed into a calculated value that the DSS can mathematically interpret accordingly. The obtained values were then used to design the final models to be solved.

#### Scenario design

The number of inputs and outputs that can be analyzed by Data Envelopment Analysis (DEA) simultaneously is limited. However, EDeS-MH is able to analyze different meaningful combinations of inputs and outputs called “scenarios” [16, 26]. Each combination was selected by experts to provide a specific and useful perspective on MH system performance. The initial group of experts included a psychologist, a health geographer, and two health engineers with experience in geospatial analysis, simulation techniques, and modeling, as well as a nurse, all of whom had previous experience in healthcare services and systems evaluation. Subsequently, a broader group of healthcare professionals who are also managers and decision-makers within the Andalusian Healthcare System—comprising medical doctors, nurses, psychologists, and others—were involved in validating the results through an iterative process. Given this, the selected input variables must be realistically related to each other and, consequently, to the output variables. There should be a reasonable causal relationship between input consumption and output production.

To describe the Andalusian MH provision from the outpatient care perspective and how acute hospital and health day care support it, four scenarios have been developed: S1) non-acute outpatient care (baseline), S2) outpatient and acute hospital care, S3) outpatient and health day care, and S4) outpatient, acute hospital and health day care (Table 2). Due to the aim of this study, services that provide rehabilitation or residential long-term care and social, education or other non-health care services were not included.

**Table 2:** Scenarios and variables identified by experts (DESDE-LTC codes are also included)

Scenario	Inputs	Outputs
S1: Non-acute Outpatient care (Baseline; DESDE codes: O9-O10)	1. Service availability in O9-O10 2. Workforce capacity (psychiatrists, nurses, psychologists and non-clinical professionals) in O9-O10	1. Prevalence in O9-O10 2. Contacts in O9-O10
S2: Baseline + acute hospital care (DESDE codes: O9-O10 + R2-O3)	1. Service availability in O9-O10 and R2-O3 2. Beds in R2 3. Workforce capacity (psychiatrists, nurses, psychologists and non-clinical professionals) in O9-O10 and R2-O3	1. Prevalence in O9-O10 2. Contacts in O9-O10
S3: Baseline + health day care (DESDE codes: O9-O10 + D1-D4.1)	1. Service availability in O9-O10 and D1-D4.1 2. Places in D1-D4.1 3. Workforce capacity (psychiatrists, nurses, psychologists and non-clinical professionals) in O9-O10 and D1-D4.1	1. Prevalence in O9-O10 2. Contacts in O9-O10
S4: Baseline + acute hospital care + health day care (DESDE codes: O9-O10 + R2-O3 + D1-D4.1)	1. Service availability in O9-O10, R2-O3 and D1-D4.1 2. Beds in R2 3. Places in D1-D4.1 4. Workforce capacity (psychiatrists, nurses, psychologists and non-clinical professionals) in O9-O10, R2-O3 and D1-D4.1	1. Prevalence in O9-O10 2. Contacts in O9-O10

#### Data envelopment analysis (DEA)

DEA is a robust non-parametric method based on linear programming, introduced by Charnes, Cooper, and Rhodes in 1978 [41] to evaluate the technical performance of a group of comparable decision-making units, in this case, each SHA. As a result of these DEA models, an indicator called Relative Technical Efficiency (RTE) is obtained and can be used to assess the performance of each unit of analysis.

For this study, DEA models were run for each scenario with variable returns to scale, considering both input orientation, which aims to minimize inputs while keeping outputs constant, and output orientation, which aims to maximize outputs while keeping inputs constant. The RTE scores were within [0, 1]: 0 denotes complete inefficiency, and 1 shows complete efficiency.

#### Descriptive and comparative statistics of RTE

The indicators to assess the performance of the system, in order to evaluate the integration of the different types of care, were RTE on average, RTE variance, statistical error (%), probability of being efficient or inefficient, probability of having a RTE score higher than 0.75, statistical stability and Shannon’s entropy. Statistical stability assesses the sensitivity (%) of RTE scores to a small change in variable values (the minimum is 0%, i.e., the system is completely unstable, and the maximum is 100%, i.e., the system is completely stable). The entropy assesses the homogeneity (%) of the RTE scores (the minimum is 0%, i.e., the distribution of the RTE scores is completely homogeneous, and the maximum is 100%, i.e., the distribution of the RTE scores is completely heterogeneous). Entropy is a common indicator for assessing how homogeneous the management of the system is.

In addition, a statistical bivariate comparison between scenarios was carried out using a Kruskal‒Wallis test with post hoc Mann‒Whitney tests using a Bonferroni-adjusted alpha level for pairwise comparisons. The effect size of the differences between groups was calculated by using the biserial correlation coefficient [42]. A statistical significance criterion of less than 5% (*p* < 0.05) was adopted for all cases.

#### Geographical visualization of RTE scores

Based on previous research results obtained in MH ecosystem assessment, an initial cutoff was determined, setting meaningful thresholds for adequate visualization of the RTE (on average) on a map [26, 35]. According to these thresholds, three cutoffs were used to geographically represent RTE scores (on average): lower than or equal to 0.75 as “low RTE”, between 0.75 and 0.9 as “medium RTE”, and higher than 0.9 as “high RTE”.

## Results

### Descriptive statistics of the SHAs

Descriptive statistics for original data are shown in Table 3.

**Table 3:** Descriptive statistics of the total number of SHAs

Variable	Average	Standard deviation	Variation coefficient (%)	Maximum	Minimum
Availability of services O9 and O10 per 100,000	1.43	0.88	61.66	5.36	0.46
Psychiatrists in O9 and O10 per 100,000	3.72	1.07	28.85	6.3	0.12
Psychologists in O9 and O10 per 100,000	2.05	0.77	37.41	5.38	0.73
Nurses in O9 and O10 per 100,000	2.08	0.78	37.29	5.32	0.05
Healthcare Assistants in O9 and O10 per 100,000	1.6	0.88	55.33	3.64	0.02
Non-clinical professionals in O9 and O10 per 100,000	2.81	1.64	58.39	10.67	0.67
Availability of services R2-O3 per 100,000	0.26	0.16	62.33	0.94	0.12
Beds in services R2-O3 per 100,000	6.73	1.87	27.81	11.31	4.32
Psychiatrists in R2-O3 per 100,000	1.08	0.29	26.6	1.92	0.77
Psychologists in R2-O3 per 100,000	0.23	0.18	79.25	0.94	0
Nurses in R2-O3 per 100,000	3.04	1.11	36.54	5.66	0
Healthcare assistants in R2-O3 per 100,000	4.19	1.43	34.05	7.54	1.93
Non-clinical professionals in R2-O3 per 100,000	1.86	1.74	93.79	9.63	0.67
Availability of services D1 and D4 per 100,000	0.47	0.11	23.23	0.68	0.33
Places in services D1 and D4 per 100,000	11.74	2.39	20.38	16.35	8.29
Psychiatrists in D1 and D4 per 100,000	0.41	0.22	54.31	0.88	0.13
Psychologists in D1 and D4 per 100,000	0.5	0.2	39.09	0.75	0.1
Nurses in D1 and D4 per 100,000	0.69	0.26	37.59	1.42	0.31
Healthcare assistants in D1 and D4 per 100,000	0.89	0.26	29.57	1.25	0.44
Non-clinical professionals in D1 and D4 per 100,000	1.83	0.54	29.67	2.55	0.78
Treated prevalence in services O9 and O10 per 1,000	20.03	5.96	29.76	35.05	4.3
Frequentation (visits) in services O9 and O10 per 1,000	91.91	37.18	40.45	208.46	5.82

### Descriptive statistics for RTE score

The DSS ran 500 simulations per SHA. The error percentage did not reach the 2.5% threshold for any cases, making the results suitable for interpretation. A summary of the descriptive statistics of DMUs’ performance from an input-oriented perspective in each scenario can be found in Table 4. Detailed descriptions of each area from both perspectives, the input-oriented and output-oriented models, are provided in tables within the supplementary files [see Additional File 2].

**Table 4:** Global RTE statistics by scenarios

Scenario	Scenario 1	Scenario 2	Scenario 3	Scenario 4
Efficiency average	0.66	0.84	0.83	0.88
Probability of being efficient	1.06%	5.38%	8.13%	12.34%
Probability of being inefficient	98.94%	94.62%	91.87%	87.66%
Probability of having an RTE greater than 0.75	21.95%	84.36%	64.94%	88.84%
Stability (%), minimum 0% maximum 100%	33.78%	46.65%	40.72%	47.43%
Shannon’s entropy (%), minimum 0% maximum 100%	71.51%	62.81%	71.07%	66.23%
Efficiency error on percentage (%)	0.26%	0.21%	0.12%	0.13%

Scenario 1, which contains outpatient care services alone (baseline), showed low global efficiency complemented by low levels of stability and high levels of entropy. These indicators highlight that outpatient care has been designed to address specific user needs that vary greatly depending on the socioeconomic structure of the corresponding SHAs.

In Scenario 2, which integrates outpatient care and acute hospital care services, the probability of being efficient, RTE on average and probability of having an RTE greater than 0.75 increased with respect to the baseline scenario (Scenario 1). This scenario also showed greater stability and lower levels of entropy. The integration of outpatient and acute hospital services had a positive effect on RTE and made the system more stable (it is more robust to changes) and homogeneous (acute hospital services have more homogenous management).

In Scenario 3, which integrates outpatient care and health day care services, the probability of being efficient, RTE on average and the probability of having an RTE greater than 0.75 also increased. This scenario showed higher stability but a similar entropy. In this case, the integration of outpatient and health day care services increased the performance and stability with respect to the baseline scenario, but the heterogeneity of the management of both types of care remained almost constant (health day case is also a “tailor-made” type of care that tries to match specific user needs).

Finally, in Scenario 4, which integrates all types of care, the probability of being efficient, RTE on average and the probability of having an RTE greater than 0.75 strongly increased. This scenario showed an intermediate stability and entropy similar to Scenarios 2 and 3. This scenario had the best performance, but it was not very stable, and the homogeneity level was relatively low.

**Fig. 2:**
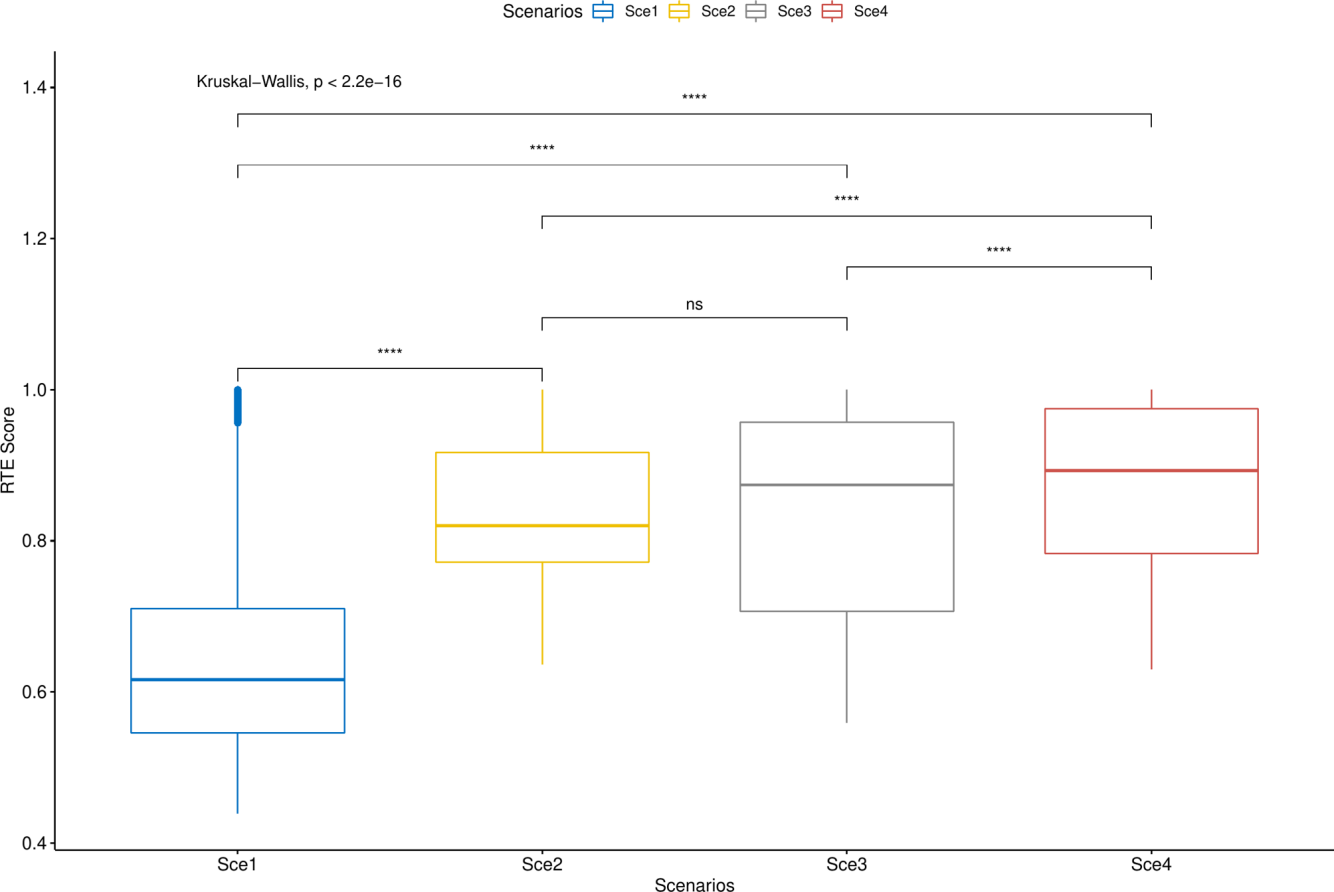
Boxplot of RTE scores for all the scenarios. **** *p* < 0.001 ^ns^ Non-significant

The Kruskal‒Wallis test highlighted statistically significant differences with a medium effect size when the RTE score was analyzed among scenarios (χ^2^ [3] = 37946.27, *p* < 0.001, η^2^_h_ = 0.292). Post hoc Mann‒Whitney tests found statistically significant differences for every comparison (*p* < 0.001 for all of them), except for Scenario 2 vs. Scenario 3 (*p* = 1). Differences between Scenario 1 and the rest of the scenarios showed a large effect size (r_bis_ = 0.983, 0.980 and 0.994). Nevertheless, differences between Scenario 4 and Scenarios 2 and 3 showed medium (r_bis_ = 0.335, for the first) and large (r_bis_ = 0.605, for the second) effect sizes. The observed RTE from the baseline scenario significantly increased when other types of care were integrated into the analysis (Fig. 2).

### Geographical distribution of RTE scores

**Fig. 3:**
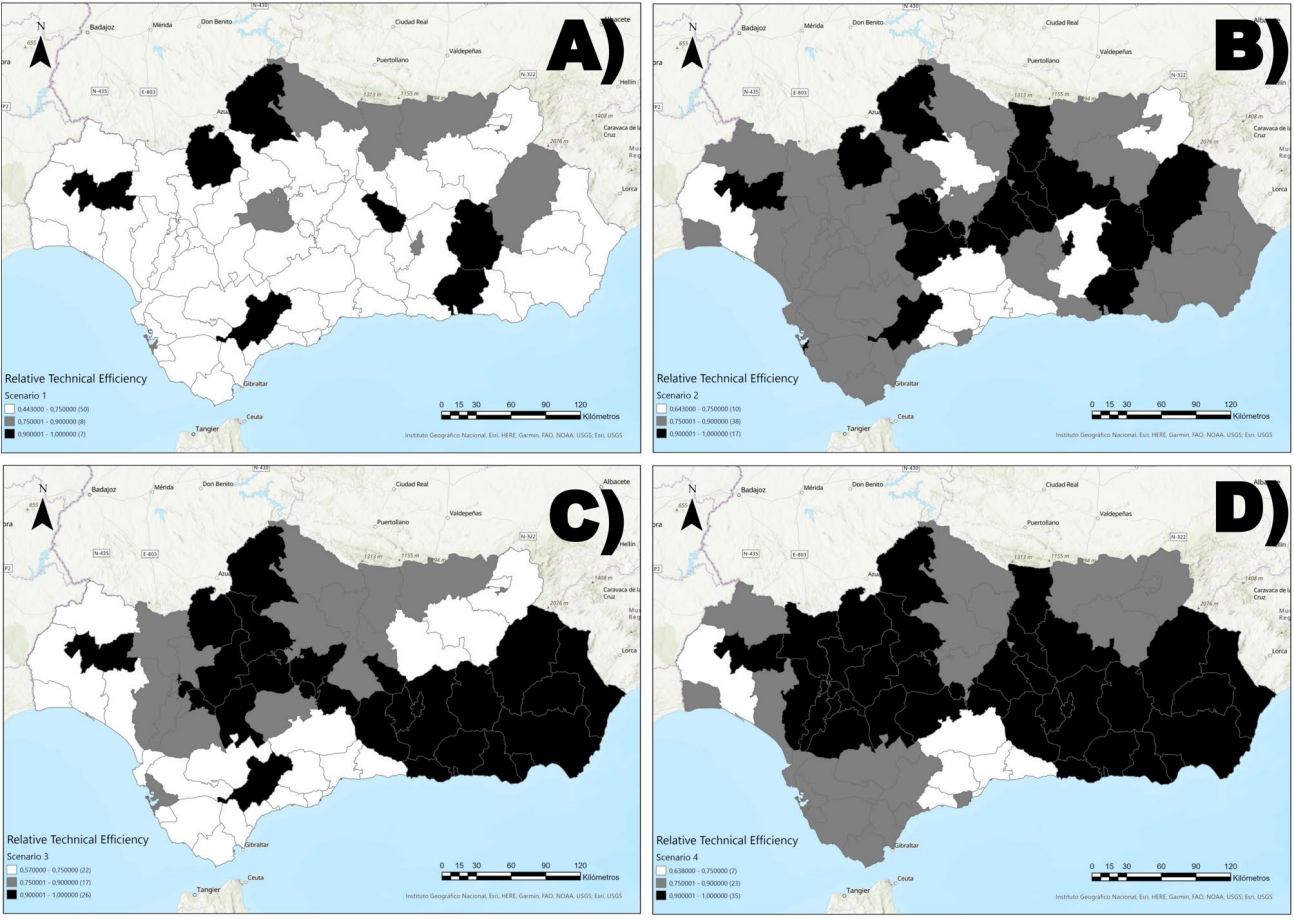
Geographical projection of RTE levels for each scenario in Andalusia. (A) Scenario 1: Outpatient care services performance; (B) Scenario 2: Outpatient and acute hospital care services performance; (C) Scenario 3: Outpatient and health day care services performance; (D) Scenario 4: Outpatient, acute hospital and health day care services performance. Darker shading means a higher RTE level on average (most efficient areas)

A high level of variability in the RTE level distribution was found among scenarios (Table 5). In Scenario 1, more than 75% of the SHAs had a low RTE (RTE on average lower than 0.75) (Fig. 3A).

**Table 5:** Number and percentages of SHAs according to their levels of RTE

RTE Level	Scenario 1	Scenario 2	Scenario 3	Scenario 4
High	7 (10.77%)	17 (26.15%)	26 (40%)	35 (53.85%)
Medium	8 (12.31%)	38 (58.46%)	17 (26.15%)	23 (35.38%)
Low	50 (76.92%)	10 (15.38%)	22 (33.85%)	7 (10.77%)

In Scenario 2, the number of SHAs with medium (in the (0.75, 0.9] RTE range) or high (RTE on average greater than 0.9) levels increased extensively, which is a behavior that continued in Scenarios 3 and 4, especially in the high RTE level. These results suggest that integrating various types of services, such as outpatient, acute hospital, and day care services, enhances the performance of the Andalusian MH system. However, the percentage of high-RTE-level SHAs is higher in Scenario 3 than in Scenario 2, indicating differences in the role of acute hospital and day care services within the system. These differences can be observed in Fig. 3B (Scenario 2), 3.C (Scenario 3) and 3.D (Scenario 4).

Scenario 4 had the highest percentage of SHAs with a high RTE level. In this scenario, it is possible to identify clusters in each category (Fig. 3D). From a global point of view, the integration of the analyzed types of care had a geographical pattern that highlights where the process either increases or does not increase the performance of the system.

## Discussion

Currently, international recommendations promote policies oriented to the design of community-based MH systems in line with the balanced care model [3]. This paradigm promotes coordinated, efficient and accessible MH care regarding population needs [19]. However, implementing these policies could present a series of difficulties and barriers [43–45], including geographic and socioeconomic barriers. For that reason, the assessment of service/area performance [22, 24, 35], the study of the potential effect of managerial interventions [26], or how balanced a MH ecosystem is [16, 46] are issues that have become relevant in the MH management research field in recent years. Furthermore, these issues coincide with a growing interest in translating the obtained results into effective and concise information in a user-friendly format for decision- and policy-makers [18]. These aspects have motivated the present study to analyze how the integration of three types of care has an impact on the performance of a real MH system both globally and geographically.

The EDeS-MH has been proven to be an appropriate tool for performance analysis of Andalusian SHAs. This methodology overcomes the limitations of the usual health care data and mainstream statistics that mainly focus on basic utilization indicators, such as the number of contacts, admissions or length of stay [47]. It also allows the inclusion of uncertainty/randomness, the application of robust procedures to diminish any lack of transparency and accountability concerning resource management [24] and, finally, the ability to obtain a more realistic picture of the MH ecosystem.

The global results exhibited a complex structure but become easier to comprehend when their geographical distribution is taken into account. Generally, the integration of the three analyzed types of care increased the performance of the system, but there were relevant differences between the Andalusian SHAs. Due to these differences, the global results of Scenarios 2 and 3 (always better than Scenario 1) slightly differed in a not very easy way to explain, except if the corresponding maps were analyzed. In some SHAs, the coordination between outpatient services and acute hospital services increased the performance better than if this coordination included health day care services. This behavior could probably be explained by differences in user needs across SHAs that MH managers tried to match appropriately. These SHAs can be identified and individually studied. From a theoretical point of view, according to the balanced care model [39], acute hospital services should have shown a strong connection to outpatient services to assure the continuity of care [48, 49]. This assumption can be confirmed in some Andalusian SHAs when other community-based types of care were included in the equation but not in all of them. Despite the expansion of community services in Andalusia since the psychiatric reform, a previous study [50] has noticed that a large proportion of high psychiatric hospitalization users remain in these specific SHAs (with acute hospital services, sometimes with more than only one), which decreased the hospital bed ratio and produces an unusual ratio of readmissions in inpatient hospitalization psychiatric units. These phenomena could be other causes of the differences between Scenarios 2 and 3. The SHAs with strong support for health day care services were mainly located in the eastern and upper center regions of Andalusia.

The results indicated that the integration of acute hospital and health day care services supported outpatient care across the region with a large effect size, producing a global improvement in MH ecosystem performance. This evidence-based finding supports the effectiveness of the theoretical model proposed by Thornicroft and Tansella [38], known as the Balanced Care Model. The model argues that efficient functioning and high-quality care are best achieved through the integration of a diverse range of services, from hospital-based to community-based care. By tailoring this integration to the region’s resources, the model aims to meet the needs of the population more effectively considering the characteristics of the real-world MH ecosystem [51]. Nevertheless, a relevant number of Andalusian SHAs were considered inefficient, mostly in the western and south-central areas. It is also relevant that both efficient and inefficient areas were geographically grouped. This characteristic spatial distribution made possible the analysis of potential differences in regional MH policy implementation, as well as in user needs.

Although both acute hospital care and health day care separately showed significant support for outpatient care raising its performance, it was actually the integration of the three types of care what increased the performance of the system.

### Implications

The present study offers an analysis of how the integration of MH types of care, as a consequence of previous Andalusian MH plans, can be related to system performance. It also identifies relevant geographical differences in Andalusian SHAs. By reviewing the reports obtained from the DSS, decision-makers can identify potential causes of the mentioned differences and propose interventions to improve areas with the weakest integration of services, thereby offering greater opportunities for improvement. Once the impact of these potential interventions is assessed, the situation can be analysed again to design new interventions and policies. This information may be useful for them, not only in the implementation of the current MH plan but also for designing the next one based on the evidence. To reach this purpose, a long-term partnership between governments and institutions with researchers can be considered critical [52]. These collaborations would be useful in designing new strategies based on expert evidence-informed knowledge, including the analysis of the potential consequences of managerial interventions in advance. Simulated and spatial data-based analyses can specifically identify potential improvements in MH care provision that always try to preserve or improve accessibility, equity and quality of care according to population needs [18, 20, 34].

### Strengths & limitations

To the best of our knowledge, this is the first study to assess the performance, in terms of RTE, of the Andalusian SHAs from a community-based point of view. The design of mixed scenarios and the application of the EDeS-MH have been shown to be suitable for assessing performance according to the balanced care model. The geographical representation of the results and the robust threshold projected for their interpretation have elicited spatial patterns of performance that could be used to identify both benchmark areas and potential areas for improvement. In addition, the EDeS-MH is a decision support system that integrates a fuzzy inference engine for interpreting variable values (previously simulated) according to a knowledge-base designed to incorporate the Balanced Care Model (local expert knowledge). This knowledge-base was structured using IF (antecedents) THEN (conclusion) rules. Simulated data values from the Monte-Carlo engine are used as the antecedents once they are interpreted by increasing or decreasing linear monotone functions (designed by expert knowledge based on the Balanced Care Model). This approach addresses the limitations of simpler methods like fuzzy ‘Benefit-of-the-Doubt’ DEA methodologies [53], which only categorize inputs and outputs as either desirable or not. Such methods are constrained by the high number of rules to be interpretated and by the use of linear functions to determine rule consequences across the entire range of variables. In contrast, our method allows for more reliable and realistic classification by defining multiple “desirable” and “undesirable” zones within the variable values range.

However, this study also has several limitations that must be acknowledged. Firstly, data collection took place during the REFINEMENT España project (2016–2018) and was cross-referenced with utilization data provided by the Andalusian Regional Government (further details are available in Additional File 1). Although this project does not directly impact the study’s aims from a methodological standpoint, it highlights the need to update the database to analyze the evolution of the MH system in Andalusia over time and to provide substantial evidence for policymakers in future research. In the coming years, these analyses will enable a comparison of the system’s performance before and after the implementation of the new Andalusian Comprehensive Mental Health and Addictions Plan (PESMA-A), which is currently being drafted to enhance coordination between the addiction and MH service networks in the region, using the standardized service description tool (DESDE-LTC). Second, two services from the dataset were excluded from the analyses because their information had missing values. This could slightly affect the results and their interpretation, although its effect should be mitigated because of the use of Monte Carlo simulation that included randomness. Third, the services included in the analysis, from the Andalusian Health Service, only include the health sector of MH care. Further studies should contemplate collecting and incorporating other care provided in the region from public and/or private institutions to highlight a complete picture of the balanced care model application [46]. From the same perspective, the inclusion of residential long-term care and social, education, or other non-health care services could identify differences not only among different areas but also elucidate more complex scenarios that can reveal different patterns of care in the region. Finally, due to the lack of information about the geographical divisions of the capitals (MH administration), the information used herein was aggregated. Thus, the granularity of the analysis was limited. Therefore, a complete and more updated dataset and administrative information from the Andalusian Regional Government is needed to overcome every limitation mentioned above. This approach could allow the assessment of the evolution of the whole ecosystem, which would help decision-makers in designing MH policies and interventions.

## Conclusion

In conclusion, the EDeS-MH decision support system has proven to be appropriate for the performance analysis of small MH areas in Andalusia according to the balanced care model. A correct implementation of appropriate types of care to match specific user needs increases the performance of the Andalusian MH system. Using data visualization tools to show the geographical distribution of SHA performance has been shown to be helpful to researchers and decision-makers in identifying patterns of service integration and spatial clusters. Both methodological approaches allowed us to identify both potential benchmark areas and areas for improvement that can be used as references for designing better policies and managerial interventions.

## Electronic supplementary material

Below is the link to the electronic supplementary material.

Supplementary Material 1

Supplementary Material 2

## Data Availability

The dataset supporting the conclusions of this article is included within the article and its additional files.

## Abbreviations

MH: Mental health
WHO: World Health Organization
SHA: Small Mental Health Area
DESDE-LTC: Description and Evaluation of Care Services and Directories for Long Term Care
RTE: Relative Technical Efficiency
DEA: Data envelopment Analysis
EDeS-MH: Decision Support System called Efficient Decision Support-MH
