# Supplementary material for: Assessment of care provision integration in a community-based mental health system: balanced care model implementation in Andalusia (Spain)
Source: BMC Public Health. 2024 Sep 30;24:2671. doi: 10.1186/s12889-024-20169-6 (PMC11440898; doi:10.1186/s12889-024-20169-6)
Supplement: Supplementary file 2 — Supplementary Material 2 [file 12889_2024_20169_MOESM2_ESM.pdf]

Supplementary Material

1 Small Mental Health Areas Location and Relative Technical Efficiency (RTE) Indicators

Supplementary Figure 1. Andalusian Small Mental Health Areas Location

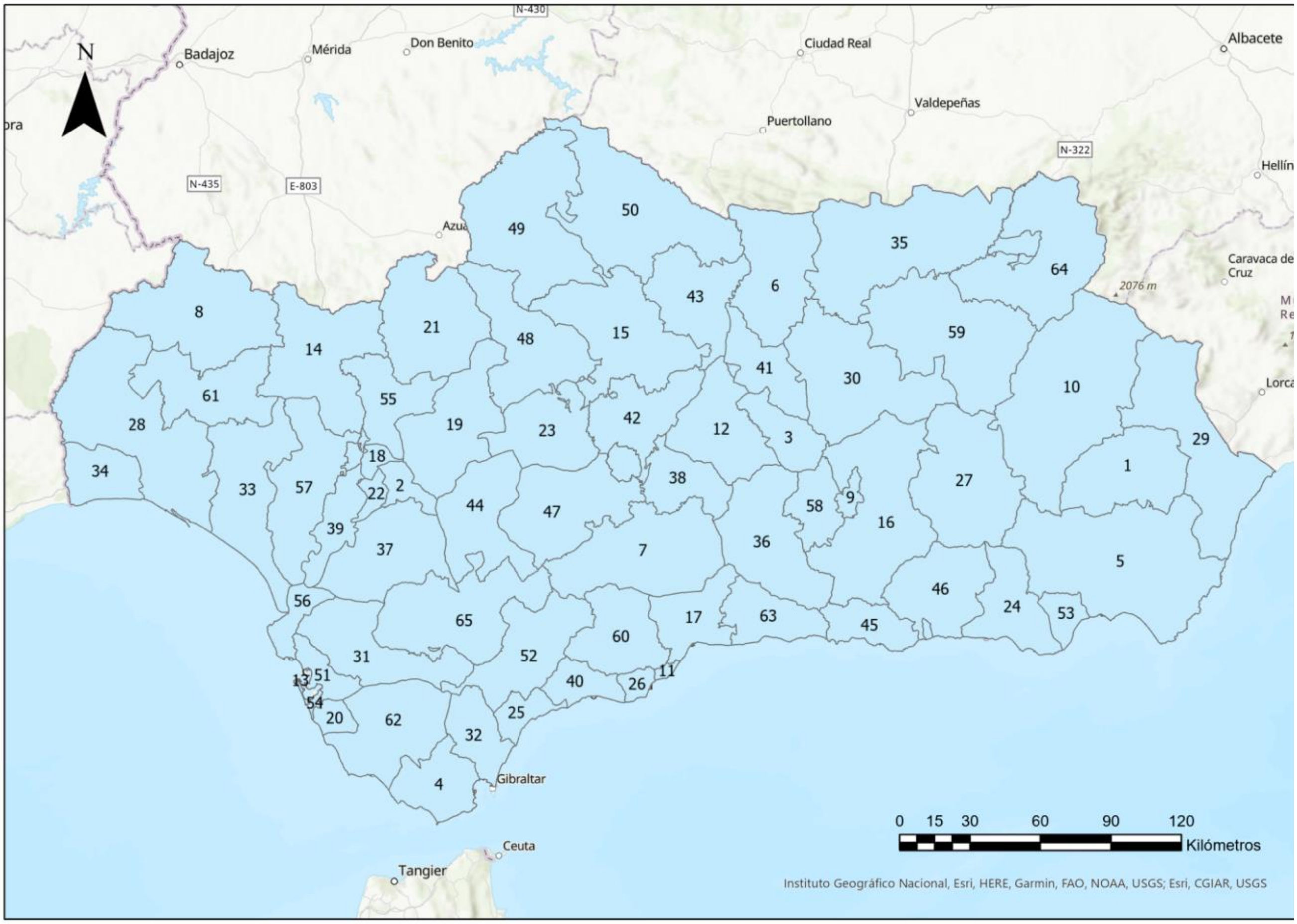

Supplementary Table 1: Descriptive statistics for RTE (Input-Oriented) score in Scenario 1

Area	Efficiency average	Efficiency error on percentage (%)	Probability of being efficient (%)	Probability of being inefficient (%)	Prob. to be higher than 75	Stability	Entropy
1	0.62	0.12	0	100	0	43.83	0
2	0.56	0.23	0	100	0	34.49	15.64
3	0.97	0.32	9	91	100	69.32	30.15
4	0.53	0.09	0	100	0	36.32	0
5	0.54	0.13	0	100	0	36.32	0
6	0.77	0.22	0	100	71.8	51.96	26.37
7	0.52	0.11	0	100	0	36.32	0
8	0.61	0.13	0	100	0	39.32	12.62
9	0.77	0.07	0	100	92.8	52.85	8.37
10	0.82	0.88	0	100	90	52.39	48.96
11	0.46	0.04	0	100	0	32.73	0
12	0.66	0.13	0	100	0	43.83	21.97
13	0.8	0.53	0	100	94	53.04	33.63
14	0.52	0.08	0	100	0	36.32	0
15	0.53	0.1	0	100	0	36.32	0
16	0.5	0.07	0	100	0	32.73	9.79
17	0.6	0.18	0	100	0	40.01	21.21
18	0.62	0.11	0	100	0	41.24	2.38
19	0.62	0.12	0	100	0	41.42	0.47
20	0.69	0.11	0	100	0	47.8	14.65
21	0.98	0.49	23.4	76.6	99.8	53.13	30.99
22	0.6	0.11	0	100	0	40.01	21.72
23	0.82	2.34	2.4	97.6	77	32.73	49.98
24	0.61	0.17	0	100	0	39	13.68
25	0.65	0.12	0	100	0	43.83	21.97
26	0.67	0.14	0	100	0	44.68	8.59
27	0.9	1.88	3.2	96.8	85.2	51.19	49.02
28	0.51	0.1	0	100	0	31.56	13.45
29	0.52	0.05	0	100	0	36.32	0
30	0.57	0.16	0	100	0	40.01	0
31	0.67	0.25	0	100	0	44.55	7.45
32	0.58	0.09	0	100	0	40.01	0
33	0.55	0.1	0	100	0	36.32	20.56
34	0.62	0.13	0	100	0	40.77	9.7
35	0.86	0.66	0	100	100	57.85	39.47
36	0.68	0.23	0	100	0	44.3	20.88
37	0.51	0.09	0	100	0	36.32	0
38	0.67	0.49	0	100	0	42.5	25.77

39	0.6	0.13	0	100	0	40.01	20.61
40	0.55	0.14	0	100	0	36.32	21.55
41	0.62	0.12	0	100	0	43.83	0
42	0.71	0.29	0	100	2.6	47.8	23.94
43	0.68	0.06	0	100	0	47.8	0.84
44	0.62	0.26	0	100	0	39.49	13.77
45	0.52	0.09	0	100	0	36.32	0
46	0.97	0.14	6.8	93.2	100	71.94	27.57
47	0.55	0.15	0	100	0	36.32	22.4
48	0.59	0.07	0	100	0	40.01	2.65
49	0.96	0.28	11	89	100	69.47	31.9
50	0.76	0.18	0	100	71	51.96	20.28
51	0.75	0.71	0	100	42.8	48.29	39.17
52	0.92	0.4	0.6	99.4	100	63.72	34.48
53	0.55	0.08	0	100	0	36.32	20.33
54	0.85	0.65	0.6	99.4	99.6	53.2	48.91
55	0.53	0.05	0	100	0	36.32	0
56	0.64	0.12	0	100	0	43.83	12.25
57	0.54	0.08	0	100	0	36.32	10.94
58	0.58	0.06	0	100	0	40.01	0
59	0.63	0.17	0	100	0	43.83	0.84
60	0.56	0.15	0	100	0	36.32	19.06
61	0.96	0.18	11.6	88.4	100	69.5	32.1
62	0.69	0.19	0	100	0	47.8	20.38
63	0.44	0.07	0	100	0	31.61	1.19
64	0.53	0.08	0	100	0	36.32	0
65	0.66	0.24	0	100	0	43.83	20.01

Supplementary Table 2: Descriptive statistics for RTE (Input-Oriented) score in Scenario 2

Area	Efficiency average	Efficiency error on percentage (%)	Probability of being efficient (%)	Probability of being inefficient (%)	Prob. to be higher than 75	Stability	Entropy
1	0.87	0.14	0	100	100	63.07	6.23
2	0.79	0.14	0	100	100	56.36	16.54
3	0.99	0.12	42.6	57.4	100	78.66	25.69
4	0.79	0.09	0	100	100	56.36	12.38
5	0.79	0.06	0	100	100	56.36	12.13
6	0.98	0.18	10.8	89.2	100	75.67	17.51
7	0.7	0.08	0	100	0	47.8	18.93
8	0.89	0.29	0	100	100	62.42	29.74
9	0.95	0.11	0	100	100	71.94	21.82
10	1	0.16	59.4	40.6	100	78.66	23.6
11	0.66	0.05	0	100	0	44.96	4.36
12	0.98	0.21	17	83	100	75.85	20.73
13	0.87	0.12	0	100	100	61	18.26
14	0.75	0.12	0	100	50.8	51.96	22.42
15	0.72	0.08	0	100	0	51.96	0
16	0.73	0.05	0	100	0	51.96	0
17	0.75	0.06	0	100	42.6	51.96	22.07
18	0.82	0.12	0	100	100	58.13	5.02
19	0.81	0.09	0	100	100	56.36	17.68
20	0.84	0.09	0	100	100	61.06	11.21
21	0.99	0.15	46.6	53.4	100	71.46	26.93
22	0.8	0.07	0	100	100	56.36	19.87
23	0.98	0.32	28.8	71.2	99	66.33	26.73
24	0.83	0.07	0	100	100	61.06	4.36
25	0.8	0.08	0	100	100	56.36	17.6
26	0.83	0.08	0	100	100	61.06	1.51
27	1	0.04	64	36	100	78.66	22.62
28	0.73	0.07	0	100	0	51.96	0
29	0.76	0.07	0	100	93.2	52.91	8.04
30	0.93	0.3	0.4	99.6	100	67.46	28.47
31	0.83	0.12	0	100	100	61.06	2.1
32	0.83	0.12	0	100	100	61.06	1.19
33	0.78	0.18	0	100	100	56.36	5.23
34	0.81	0.14	0	100	100	55.39	17.15
35	0.87	0.3	0	100	100	61.06	36.75
36	0.84	0.11	0	100	100	61.06	15.73
37	0.76	0.09	0	100	95.4	53.27	6.04
38	0.97	0.2	13.8	86.2	100	74.69	24.99

39	0.8	0.1	0	100	100	56.36	22.07
40	0.73	0.08	0	100	0	51.96	0
41	0.93	0.27	1.6	98.4	100	67.36	30.63
42	0.83	0.09	0	100	100	61.06	4.8
43	0.83	0.15	0	100	100	61.06	4.58
44	0.83	0.09	0	100	100	61.06	5.84
45	0.75	0.11	0	100	60.8	51.96	21.66
46	0.98	0.05	13.4	86.6	100	76.25	14.67
47	0.97	0.76	9.6	90.4	99.4	64.2	30.06
48	0.78	0.08	0	100	100	56.36	0
49	0.99	0.1	20	80	100	78.66	20.12
50	0.88	0.09	0	100	100	66.2	1.81
51	0.9	0.25	0	100	100	66.2	22.3
52	0.94	0.33	0.6	99.4	100	68.98	24.18
53	0.79	0.1	0	100	100	56.36	4.36
54	0.94	0.28	0.8	99.2	100	68.43	28.72
55	0.76	0.08	0	100	98	53.67	3.17
56	0.79	0.11	0	100	100	56.36	13.56
57	0.77	0.08	0	100	100	56.36	0
58	0.8	0.07	0	100	100	56.36	17.9
59	0.79	0.12	0	100	100	56.36	15.15
60	0.73	0.07	0	100	0.2	51.96	0.47
61	0.98	0.24	20.4	79.6	100	75.19	26.27
62	0.82	0.1	0	100	100	61.06	0
63	0.64	0.05	0	100	0	43.83	4.58
64	0.75	0.11	0	100	43.8	51.96	22.18
65	0.82	0.15	0	100	100	58.56	1.51

Supplementary Table 3: Descriptive statistics for RTE (Input-Oriented) score in Scenario 3

Area	Efficiency average	Efficiency error on percentage (%)	Probability of being efficient (%)	Probability of being inefficient (%)	Prob. to be higher than 75	Stability	Entropy
1	0.99	0.3	43	57	100	72.9	31.06
2	0.9	0.61	3.4	96.6	99.4	55.84	52.02
3	0.98	0.13	25.4	74.6	100	75.48	26.13
4	0.63	0.08	0	100	0	43.83	1.19
5	0.98	0.31	29.4	70.6	100	67.58	31.34
6	0.88	0.16	0	100	100	63.2	16.09
7	0.62	0.07	0	100	0	43.83	0
8	0.63	0.13	0	100	0	43.83	4.58
9	0.98	0.15	29.2	70.8	100	75.82	26.24
10	0.98	0.31	31.6	68.4	100	74.83	30.29
11	0.57	0.03	0	100	0	40.01	0
12	0.88	1.15	1.8	98.2	100	56.36	52.5
13	0.85	0.17	0	100	100	58.62	23.7
14	0.89	1.37	1	99	88.4	50.85	51.79
15	0.82	0.76	0.4	99.6	74.8	47.06	62.33
16	0.9	0.76	3.8	96.2	100	62.06	43.66
17	0.7	0.09	0	100	0	47.8	19.98
18	0.91	0.71	4.4	95.6	100	58.44	50.48
19	0.91	0.88	7.2	92.8	100	58.37	53.39
20	0.74	0.16	0	100	22.6	51.96	17.29
21	1	0.17	66.6	33.4	100	76.19	23.65
22	0.89	0.63	1.4	98.6	94	53.49	52.29
23	0.95	0.54	15.4	84.6	96.2	60.59	43.56
24	0.98	0.37	37	63	100	70.43	32.38
25	0.69	0.13	0	100	0	47.8	16.8
26	0.7	0.13	0	100	0	47.8	21.58
27	0.98	0.53	30	70	100	71.56	35.51
28	0.61	0.1	0	100	0	38.64	14.75
29	0.97	0.44	15	85	100	69.38	31.25
30	0.72	0.16	0	100	0	49.49	1.19
31	0.71	0.16	0	100	0	47.8	19.82
32	0.68	0.15	0	100	0	47.8	0
33	0.66	0.1	0	100	0	43.83	20.08
34	0.71	0.11	0	100	0	48.19	9.64
35	0.9	0.42	0	100	100	63.39	32.13
36	0.98	0.27	26	74	100	73.51	32.44
37	0.88	0.75	1.8	98.2	88.6	50.89	52.61
38	0.92	0.45	4.4	95.6	100	58.51	48.83

39	0.9	0.52	2	98	95.4	55.87	52.4
40	0.63	0.07	0	100	0	43.83	0
41	0.77	0.14	0	100	97	53.52	4.82
42	0.93	0.53	4.4	95.6	100	63.2	42.94
43	0.86	0.75	2	98	100	56.36	53.14
44	0.93	0.66	8.4	91.6	100	59.4	49.22
45	0.91	0.23	1.4	98.6	100	62.19	42.91
46	0.99	0.09	49.4	50.6	100	76.1	25.84
47	0.88	0.66	1	99	88	52.05	52.46
48	0.9	0.74	3.2	96.8	100	58.25	50.4
49	0.98	0.21	30.4	69.6	100	75.25	30.02
50	0.9	0.69	2.4	97.6	100	61.11	44.43
51	0.86	0.37	0	100	100	58.59	29.41
52	0.91	0.23	0.6	99.4	100	63.48	34.65
53	0.97	0.25	21	79	100	68.85	32.54
54	0.89	0.46	1.2	98.8	100	61.54	39.77
55	0.88	0.94	0.6	99.4	85.4	49.21	52.02
56	0.68	0.09	0	100	0	47.8	0
57	0.89	1.44	1.8	98.2	87.2	49.53	53.4
58	0.9	0.36	2.2	97.8	100	62.42	42.32
59	0.71	0.1	0	100	0	47.8	17.6
60	0.64	0.08	0	100	0	43.83	2.38
61	0.97	0.21	18.2	81.8	100	69.5	34.76
62	0.72	0.13	0	100	0	49.55	0.47
63	0.57	0.06	0	100	0	40.01	0
64	0.63	0.1	0	100	0	43.83	0
65	0.73	0.09	0	100	3.8	51.96	5.23

Supplementary Table 4: Descriptive statistics for RTE (Input-Oriented) score in Scenario 4

Area	Efficiency average	Efficiency error on percentage (%)	Probability of being efficient (%)	Probability of being inefficient (%)	Prob. to be higher than 75	Stability	Entropy
1	0.99	0.1	53.4	46.6	100	76.25	25.43
2	0.91	0.5	1.6	98.4	100	61.54	43.54
3	0.99	0.08	53.2	46.8	100	78.66	25.23
4	0.78	0.08	0	100	100	56.36	1.19
5	0.99	0.19	36.2	63.8	100	75.89	27.09
6	0.96	0.31	6.4	93.6	100	71.94	27.06
7	0.68	0.05	0	100	0	47.8	0
8	0.84	0.23	0	100	100	58.38	23.4
9	0.99	0.15	39.4	60.6	100	76.25	24.97
10	1	0.09	75.4	24.6	100	78.66	20.59
11	0.65	0.03	0	100	0	43.83	22.37
12	0.98	0.12	11.6	88.4	100	75.51	18.98
13	0.87	0.17	0	100	100	61.99	17.52
14	0.9	0.77	1.6	98.4	100	58.13	46.59
15	0.77	0.07	0	100	100	56.36	0
16	0.92	0.3	2	98	100	63.72	35.93
17	0.73	0.08	0	100	0.4	51.96	0.84
18	0.93	0.48	4.8	95.2	100	64.22	37.37
19	0.92	0.58	5	95	100	61.26	45.61
20	0.77	0.07	0	100	99.8	53.95	0.47
21	1	0.07	65.4	34.6	100	76.16	23.98
22	0.91	0.42	0.8	99.2	100	60.78	44.55
23	0.99	0.18	59	41	100	68.99	26.48
24	0.99	0.19	44.6	55.4	100	76.1	26.66
25	0.77	0.07	0	100	100	56.36	0
26	0.76	0.04	0	100	88.2	52.08	11.74
27	1	0.06	84.8	15.2	100	82.51	15.15
28	0.72	0.05	0	100	0	49.55	0.47
29	0.98	0.16	22.8	77.2	100	72.97	27.42
30	0.95	0.34	2.6	97.4	100	69.35	28.1
31	0.81	0.06	0	100	100	56.36	19.82
32	0.8	0.12	0	100	100	56.36	22.37
33	0.77	0.09	0	100	100	56.36	0
34	0.79	0.16	0	100	100	56.36	19.12
35	0.85	0.37	0.2	99.8	100	58.65	25.4
36	0.98	0.19	24.8	75.2	100	75.45	26.37
37	0.9	0.43	0.8	99.2	100	58.16	47.63
38	0.98	0.17	14.8	85.2	100	75.67	19.93
39	0.92	0.43	1.8	98.2	100	62.32	43.4

40	0.69	0.07	0	100	0	47.8	6.98
41	0.95	0.34	2.4	97.6	100	69.44	27.21
42	0.85	0.1	0	100	100	61.06	24.16
43	0.85	0.11	0	100	100	61.06	21.8
44	0.94	0.43	7.8	92.2	100	62.85	44.8
45	0.93	0.31	2.6	97.4	100	66.9	33.86
46	0.99	0.11	51.2	48.8	100	78.66	25.12
47	0.98	0.22	32.2	67.8	100	68.4	29.93
48	0.91	0.6	4.8	95.2	100	58.56	47.72
49	0.99	0.06	35.8	64.2	100	78.66	23.98
50	0.89	0.05	0	100	100	66.2	14.75
51	0.85	0.17	0	100	100	61.06	25.02
52	0.81	0.19	0	100	100	57.06	10.66
53	0.98	0.16	24.4	75.6	100	75.22	25.81
54	0.9	0.41	1.2	98.8	100	63.14	35.82
55	0.91	0.33	2.2	97.8	100	58.69	44.66
56	0.78	0.1	0	100	100	56.36	4.13
57	0.91	0.68	1.4	98.6	100	61.04	44.08
58	0.93	0.27	2	98	100	66.2	34.04
59	0.78	0.06	0	100	100	56.36	0.47
60	0.68	0.07	0	100	0	47.8	0
61	0.98	0.19	20.8	79.2	100	74.89	26.6
62	0.8	0.1	0	100	100	56.36	21.85
63	0.64	0.09	0	100	0	43.83	0
64	0.76	0.1	0	100	86	51.7	13.1
65	0.78	0.08	0	100	100	56.36	0

Supplementary Table 5: Descriptive statistics for RTE (Output-Oriented) score in Scenario 1

Area	Efficiency average	Efficiency error on percentage (%)	Probability of being efficient (%)	Probability of being inefficient (%)	Prob. to be higher than 75	Stability	Entropy
Global	0.606	0.332083	1.06	98.94	22.15	13.2045	86
1	0.59	0.284234	0	100	0	40.0142	18.67
2	0.645	0.208433	0	100	0	43.8331	21.99
3	0.967	0.678771	9	91	100	69.1021	31.92
4	0.535	0.209142	0	100	0	36.3206	12.5
5	0.509	0.509031	0	100	0	32.7321	19.18
6	0.665	0.463256	0	100	0	43.8331	28.14
7	0.146	0.247332	0	100	0	9.5663	15.64
8	0.626	0.32903	0	100	0	39.9629	23.24
9	0.343	0.252593	0	100	0	22.4566	17.98
10	0.652	0.493161	0	100	0.8	40.7417	35.76
11	0.788	0.413813	0	100	91.2	52.5863	31.02
12	0.202	0.161186	0	100	0	12.7211	17.29
13	0.698	0.554271	0	100	0	47.8036	22.09
14	0.837	0.235272	0	100	100	58.0353	25.48
15	0.565	0.286631	0	100	0	34.4169	19.32
16	0.277	0.059969	0	100	0	19.161	0
17	0.533	0.549829	0	100	0	36.3206	14.95
18	0.695	0.251025	0	100	0.4	47.8036	21.89
19	0.454	0.138516	0	100	0	31.614	21.87
20	0.645	0.328099	0	100	0	43.8331	21.48
21	0.533	0.136938	0	100	0	36.3206	9.33
22	0.965	0.524665	23.4	76.6	99.8	57.5052	36.65
23	0.52	0.36917	0	100	0	33.0382	11.46
24	0.808	2.290045	2.4	97.6	77	34.3795	57.6
25	0.43	0.176887	0	100	0	31.614	6.04
26	0.133	0.031124	0	100	0	9.5663	0
27	0.302	0.596037	0	100	0	19.161	22.07
28	0.926	3.382298	3.2	96.8	88.2	54.3281	41.57
29	0.49	0.190796	0	100	0	32.7321	19.12
30	0.446	0.235736	0	100	0	31.614	21.06
31	0.632	0.524213	0	100	0	40.9926	19.88
32	0.59	0.318185	0	100	0	40.0142	18.47
33	0.56	0.236466	0	100	0	36.3206	19.42
34	0.559	0.240476	0	100	0	36.3206	21.03
35	0.638	0.400886	0	100	0	41.3917	19.67
36	0.935	0.255746	0	100	100	69.226	20.68
37	0.752	0.247633	0	100	51.2	51.9615	24.26

38	0.85	0.440298	0	100	100	61.0636	26.04
39	0.522	0.137983	0	100	0	33.134	13.31
40	0.365	0.225214	0	100	0	22.4325	7.69
41	0.631	0.646737	0	100	0	41.0547	19.08
42	0.772	0.414694	0	100	80.6	50.7223	29.18
43	0.613	0.297063	0	100	0	40.0142	21.94
44	0.711	0.343334	0	100	5.2	47.8036	26.84
45	0.445	0.193073	0	100	0	31.614	20.33
46	0.953	0.438274	6.8	93.2	100	69.4708	31.28
47	0.562	0.28512	0	100	0	36.3206	19.26
48	0.582	0.119077	0	100	0	37.5728	15.57
49	0.925	1.222012	11	89	100	59.8437	49.2
50	0.748	0.619132	0	100	46.6	49.3984	28.15
51	0.929	0.29156	0.6	99.4	100	67.0029	31.51
52	0.342	0.086768	0	100	0	22.4566	9.79
53	0.845	0.795575	0.6	99.4	98.8	53.7939	49.27
54	0.537	0.388271	0	100	0	36.3206	14.12
55	0.537	0.175739	0	100	0	36.3206	13.9
56	0.487	0.150569	0	100	0	32.7321	15.64
57	0.531	0.246928	0	100	0	36.3206	12.13
58	0.528	0.160651	0	100	0	33.7269	13.27
59	0.577	0.277381	0	100	0	37.0802	18.5
60	0.449	0.19146	0	100	0	31.614	22.3
61	0.967	0.3177	11.6	88.4	100	71.9426	30.21
62	0.644	0.283092	0	100	0	43.8331	20.86
63	0.598	0.200602	0	100	0	40.0142	21.8
64	0.505	0.251794	0	100	0	32.7321	21.77
65	0.643	0.228025	0	100	0	43.8331	21.18

Supplementary Table 6: Descriptive statistics for RTE (Output-Oriented) score in Scenario 2

Area	Efficiency average	Efficiency error on percentage (%)	Probability of being efficient (%)	Probability of being inefficient (%)	Prob. to be higher than 75	Stability	Entropy
Global	0.631	0.305983	5.38	94.62	27.85	13.4433	87.54
1	0.622	0.229085	0	100	0	38.7497	26.23
2	0.678	0.155269	0	100	0	44.1647	23.26
3	0.984	0.303863	42.6	57.4	100	72.6766	33.76
4	0.549	0.272611	0	100	0	36.3206	22.25
5	0.53	0.326808	0	100	0	33.7575	13.13
6	0.85	0.382736	10.8	89.2	92.2	50.3699	57.75
7	0.146	0.305432	0	100	0	9.5663	16.19
8	0.631	0.26537	0	100	0	40.227	25.98
9	0.346	0.371124	0	100	0	22.4566	21.02
10	0.656	0.433216	0	100	0.4	40.9926	35.65
11	0.984	0.222121	59.4	40.6	100	74.1122	30.85
12	0.203	0.103683	0	100	0	11.505	12.25
13	0.927	0.668806	17	83	100	60.7228	47.57
14	0.836	0.29223	0	100	100	57.7491	26.44
15	0.567	0.111895	0	100	0	35.2169	18.59
16	0.281	0.070985	0	100	0	19.161	0
17	0.532	0.62807	0	100	0	36.3206	14.12
18	0.693	0.607752	0	100	0	47.8036	20.74
19	0.456	0.238297	0	100	0	31.614	21.14
20	0.647	0.370472	0	100	0	43.8331	22.03
21	0.534	0.148416	0	100	0	36.3206	9.18
22	0.976	0.549407	46.6	53.4	99.6	59.8557	36.5
23	0.543	0.304674	0	100	0	36.3206	20.91
24	0.95	0.822714	28.8	71.2	98.8	53.4499	45.97
25	0.446	0.179448	0	100	0	31.614	20.95
26	0.133	0.030865	0	100	0	9.5663	0
27	0.304	0.61996	0	100	0	19.161	20.99
28	0.989	0.770983	64	36	100	73.1279	27.71
29	0.49	0.189247	0	100	0	32.7321	18.61
30	0.465	0.382268	0	100	0	30.6657	13.54
31	0.639	0.602501	0.4	99.6	1.2	41.1165	25.54
32	0.593	0.271511	0	100	0	40.0142	20.13
33	0.578	0.210023	0	100	0	37.1114	17.02
34	0.556	0.248309	0	100	0	36.3206	21.39
35	0.638	0.360965	0	100	0	41.27	21.45
36	0.935	0.321056	0	100	100	69.2568	20.67
37	0.751	0.258984	0	100	49.8	51.9615	24.25

38	0.95	0.785159	13.8	86.2	100	68.6922	38.28
39	0.522	0.136561	0	100	0	33.1975	13.12
40	0.365	0.147709	0	100	0	22.6247	6.61
41	0.646	1.377904	1.6	98.4	3.6	41.0857	30.4
42	0.771	0.211803	0	100	79.6	51.9615	29.02
43	0.612	0.248632	0	100	0	40.0142	23.7
44	0.746	0.555703	0	100	42	49.4594	25.98
45	0.446	0.119446	0	100	0	31.614	21.18
46	0.955	0.591493	13.4	86.6	100	69.3489	35.15
47	0.912	2.062299	9.6	90.4	95.4	52.9667	50.95
48	0.583	0.15642	0	100	0	37.5121	17.09
49	0.938	0.42388	20	80	100	63.632	45.03
50	0.75	0.580818	0	100	49	49.4289	27.48
51	0.928	0.458594	0.6	99.4	100	67.0742	30.89
52	0.35	0.096535	0	100	0	22.4566	22.18
53	0.851	0.675761	0.8	99.2	98.6	53.3811	49.28
54	0.546	0.390945	0	100	0	36.3206	21.55
55	0.537	0.154611	0	100	0	36.3206	12.86
56	0.488	0.161666	0	100	0	32.7321	17.05
57	0.531	0.388913	0	100	0	33.9094	11.67
58	0.536	0.309175	0	100	0	36.3206	17.21
59	0.604	0.209813	0	100	0	40.0142	22.68
60	0.448	0.26908	0	100	0	31.614	21.85
61	0.973	0.470479	20.4	79.6	100	73.2519	32.84
62	0.643	0.231789	0	100	0	43.8331	20.65
63	0.597	0.227039	0	100	0	40.0142	21.39
64	0.525	0.243196	0	100	0	33.2923	15.44
65	0.645	0.241414	0	100	0	43.8331	21.32

Supplementary Table 7: Descriptive statistics for RTE (Output-Oriented) score in Scenario 3

Area	Efficiency average	Efficiency error on percentage (%)	Probability of being efficient (%)	Probability of being inefficient (%)	Prob. to be higher than 75	Stability	Entropy
Global	0.710	0.373828	8.12	91.88	43.24	15.3839	87.03
1	0.963	0.418287	43	57	99.8	61.0291	42.36
2	0.874	0.494258	3.4	96.6	99.8	54.945	47.85
3	0.978	0.230213	25.4	74.6	100	71.87	32.71
4	0.533	0.249779	0	100	0	36.3206	10.94
5	0.94	0.64606	29.4	70.6	99	50.5528	52.58
6	0.673	0.306221	0	100	0.2	42.3179	28.17
7	0.147	0.401631	0	100	0	9.5663	16.01
8	0.627	0.364319	0	100	0	40.227	21.89
9	0.794	1.914841	29.2	70.8	53	37.4817	67.24
10	0.651	0.491987	0	100	1.4	40.3248	38.64
11	0.97	0.440522	31.6	68.4	100	69.4404	36.13
12	0.206	0.165347	0	100	0	13.1084	4.13
13	0.847	0.947439	1.8	98.2	80.2	48.2663	54.94
14	0.836	0.263085	0	100	100	57.4574	29.19
15	0.758	0.63667	1	99	57.8	45.1678	47.49
16	0.375	0.853638	0.4	99.6	0.4	28.6848	45.86
17	0.696	1.613194	3.8	96.2	7	43.9998	35.34
18	0.695	0.310887	0	100	0	47.8036	21.85
19	0.65	1.049018	4.4	95.6	8.6	36.2766	57
20	0.873	1.309206	7.2	92.8	97	53.4906	56.98
21	0.533	0.118833	0	100	0	36.3206	10.37
22	0.988	0.46976	66.6	33.4	100	70.3935	27.99
23	0.71	0.507495	1.4	98.6	17.8	40.8363	48.59
24	0.875	1.254923	15.4	84.6	81.8	43.8024	65.51
25	0.902	0.881788	37	63	91.8	44.1474	60.31
26	0.132	0.023157	0	100	0	9.5663	0
27	0.305	0.630497	0	100	0	19.161	18.87
28	0.971	1.458837	30	70	100	66.2559	40.01
29	0.495	0.178648	0	100	0	32.7321	22.03
30	0.83	1.974847	14.6	85.4	85.4	44.4522	57.91
31	0.637	0.279117	0	100	0	41.1165	22.06
32	0.594	0.26353	0	100	0	40.0142	20.67
33	0.558	0.397957	0	100	0	36.3206	21.63
34	0.561	0.271385	0	100	0	36.3206	21.46
35	0.649	0.445986	0	100	0	43.8331	23.78
36	0.94	0.197307	0	100	100	69.2568	22.75
37	0.963	0.295701	26	74	100	69.3183	37.9

38	0.929	0.281544	4.4	95.6	100	63.0085	43.65
39	0.713	0.60828	2	98	18.6	41.0099	48.95
40	0.365	0.136781	0	100	0	22.5609	6.98
41	0.637	0.732958	0	100	0	41.3917	19.48
42	0.909	1.060303	4.4	95.6	99.6	55.8732	50.19
43	0.749	0.849259	2	98	61	41.27	52.93
44	0.927	0.477675	8.4	91.6	100	63.0085	44.47
45	0.572	0.73076	1.4	98.6	2	36.3206	26.11
46	0.981	0.372908	49.4	50.6	100	73.7597	32.88
47	0.745	0.61281	1	99	46	44.2056	45.91
48	0.785	0.464339	3.2	96.8	67.8	48.2569	53.92
49	0.958	0.679412	30.2	69.8	100	63.143	45.91
50	0.869	0.93452	2.4	97.6	92.6	52.8162	53.24
51	0.929	0.407852	0.6	99.4	100	67.1452	30.86
52	0.777	1.423102	21	79	48.6	36.0394	66.15
53	0.845	0.560195	1.2	98.8	99	53.8244	48.7
54	0.723	0.730568	0.6	99.4	26.6	43.6984	49.75
55	0.534	0.152966	0	100	0	36.3206	9.79
56	0.657	0.709385	1.8	98.2	4	36.4408	46.81
57	0.684	0.849921	2.2	97.8	3.4	43.6984	28.77
58	0.532	0.404432	0	100	0	33.7269	17.25
59	0.763	0.498407	1.8	98.2	60.6	45.3394	46.75
60	0.45	0.15286	0	100	0	31.614	22.38
61	0.97	0.432674	18.2	81.8	100	71.9426	32.97
62	0.639	0.306805	0	100	0	43.8331	16.71
63	0.598	0.254024	0	100	0	40.0142	21.94
64	0.539	0.286969	0	100	0	36.3206	18.26
65	0.644	0.247391	0	100	0	43.8331	21.51

Supplementary Table 8: Descriptive statistics for RTE (Output-Oriented) score in Scenario 4

Area	Efficiency average	Efficiency error on percentage (%)	Probability of being efficient (%)	Probability of being inefficient (%)	Prob. to be higher than 75	Stability	Entropy
Global	0.708	0.455351	12.28	87.72	42.55	15.3279	86.3
1	0.974	0.392234	53.6	46.4	100	66.5962	37.18
2	0.835	0.784235	1.4	98.6	84.6	49.0721	54.39
3	0.986	0.127867	52.6	47.4	100	72.7093	31.55
4	0.545	0.231866	0	100	0	36.3206	20.86
5	0.949	0.444292	35.6	64.4	100	60.7859	47.57
6	0.647	1.025841	6.4	93.6	7	39.0357	31.66
7	0.146	0.266789	0	100	0	9.5663	15.82
8	0.629	0.232595	0	100	0	40.227	24.21
9	0.825	1.541598	39.4	60.6	58.6	38.1377	60.96
10	0.656	0.358373	0	100	0.6	40.5827	38.68
11	0.991	0.151772	77.2	22.8	100	72.7745	22.61
12	0.207	0.221413	0	100	0	13.3852	1.81
13	0.881	0.985858	9.8	90.2	100	57.717	51.48
14	0.835	0.289767	0	100	100	57.7491	25.72
15	0.738	0.997069	1.4	98.6	43.6	40.5187	52.06
16	0.287	0.084808	0	100	0	19.161	1.51
17	0.69	1.419792	2	98	4.8	43.6984	34.41
18	0.691	0.155282	0	100	0	45.3622	20.16
19	0.649	1.250871	4.2	95.8	12.8	32.4413	65.27
20	0.865	1.857325	4.6	95.4	83.8	50.8899	58.48
21	0.536	0.111975	0	100	0	36.3206	11.61
22	0.985	0.494355	62.2	37.8	100	67.1081	30.85
23	0.694	0.74545	0.8	99.2	13.6	36.8658	52.24
24	0.974	0.763515	59.2	40.8	99.2	54.3424	36.95
25	0.92	0.913434	46.2	53.8	94.8	47.2986	53.53
26	0.133	0.013868	0	100	0	9.5663	0
27	0.305	0.779221	0	100	0	19.161	19.48
28	0.995	0.526632	84.4	15.6	100	77.4959	17.89
29	0.498	0.188593	0	100	0	32.7321	22.31
30	0.834	1.833756	21.4	78.6	86.8	44.6975	50.66
31	0.653	0.846016	3	97	4.2	41.2394	30.21
32	0.592	0.160897	0	100	0	40.0142	19.87
33	0.576	0.432106	0	100	0	36.6355	18.78
34	0.563	0.279857	0	100	0	36.3206	22.54
35	0.652	0.249388	0	100	0	43.8331	26.95
36	0.937	0.239168	0.2	99.8	100	69.2876	21.66
37	0.963	0.42136	24.8	75.2	100	69.1021	38.36
38	0.953	0.324389	15.6	84.4	100	69.1951	36.36

39	0.697	0.896819	1.4	98.6	17.4	36.3326	54.39
40	0.365	0.145847	0	100	0	22.1723	9.02
41	0.651	0.938352	3.2	96.8	3.8	41.27	28.22
42	0.788	0.286617	0	100	95.8	53.3287	26.94
43	0.627	0.226034	0	100	0	40.8677	15.04
44	0.911	1.106846	8.2	91.8	99.8	55.8732	53.78
45	0.579	0.825189	2.6	97.4	3.2	36.3206	27.11
46	0.983	0.351354	53.8	46.2	100	71.8354	32.7
47	0.951	0.824363	31.8	68.2	99.2	55.7283	47.52
48	0.795	0.653316	4.8	95.2	70.8	44.0205	65.37
49	0.951	0.593559	35.2	64.8	100	61.8792	45.79
50	0.755	0.281924	0	100	57.2	49.4898	27.63
51	0.834	0.249073	0	100	100	58.4086	18.05
52	0.738	2.202854	23.2	76.8	28.2	37.1769	55.75
53	0.851	0.607321	1.2	98.8	98.8	53.6353	48.9
54	0.719	1.1246	2.2	97.8	27.2	39.7879	54.25
55	0.537	0.060292	0	100	0	36.3206	12.38
56	0.649	0.602286	1.4	98.6	3.6	36.0035	49.98
57	0.684	1.175334	2	98	3.8	43.5625	31
58	0.538	0.26705	0	100	0	36.3206	18.4
59	0.761	1.010941	0.6	99.4	62.6	43.9832	54.01
60	0.451	0.19072	0	100	0	31.614	22.39
61	0.972	0.227957	20.8	79.2	100	72.9539	32.85
62	0.645	0.275677	0	100	0	43.8331	20.91
63	0.595	0.237802	0	100	0	40.0142	20.7
64	0.544	0.425461	0	100	0	36.3206	21.32
65	0.641	0.231152	0	100	0	43.8331	20.86
